# Provision of rehabilitation for congenital conditions

**DOI:** 10.2471/BLT.22.288147

**Published:** 2022-09-19

**Authors:** Tracey Smythe, Lindsey Freeze, Anna Cuthel, Michelle Flowers, Frederic Seghers, Nukhba Zia, Abdulgafoor M Bachani

**Affiliations:** a International Centre for Evidence in Disability, London School of Hygiene & Tropical Medicine, Keppel Street, London WC1E 7HT, England.; b MiracleFeet, Chapel Hill, United States of America (USA).; c KwaZulu-Natal, South Africa.; d Clinton Health Access Initiative, Boston, USA.; e Department of International Health, Johns Hopkins Bloomberg School of Public Health, Baltimore, USA.

## Abstract

Considerable progress has been made in saving the lives of children younger than 5 years. Nevertheless, these advances have failed to help all children thrive, particularly children with disabilities. We describe the increasing prevalence of disability among children and adolescents. We evaluate the current situation regarding children with disabilities and rehabilitation in the context of health systems, particularly those in low- and middle-income countries. Within the newborn health agenda, congenital anomalies often require early intervention and rehabilitation. We provide Argentina as an example of a country where rehabilitation for congenital anomalies is integrated into the health system. We argue that congenital anomalies that require rehabilitation have the potential to strengthen rehabilitation systems and policies by: strengthening coordination between primary care and rehabilitation; identifying and understanding pathways that allow families to engage with services; providing human resources for rehabilitation; and building systems and resources that support assistive technology and rehabilitation. We propose ways for countries to prioritize and integrate early identification, referral and care for children with congenital anomalies to strengthen health systems for all. We identify opportunities to expand policy and planning and to design service delivery and workforce strategies through World Health Organization guidelines and frameworks for rehabilitation. We argue that the global health community must act to ensure that rehabilitation services to support functioning from birth are well established, accepted and integrated within health systems, and that disability is prioritized within child health. These steps would strengthen health systems, ensure functioning from birth and make rehabilitation accessible to all.

## Résumé

Des progrès considérables ont été réalisés pour sauver la vie des enfants de moins de cinq ans. Pourtant, ces avancées n'ont pas permis à tous les enfants de s'épanouir, en particulier lorsqu'ils présentent un handicap. Dans ce document, nous faisons état de la prévalence croissante du handicap chez les enfants et adolescents. Nous évaluons la situation actuelle des enfants porteurs de handicap ainsi que leur réadaptation au sein des systèmes de santé, surtout dans les pays à revenu faible et intermédiaire. Dans le cadre du programme de santé néonatale, les anomalies congénitales nécessitent souvent des interventions et une réadaptation rapides. Nous utilisons l'exemple de l'Argentine, un pays où la réadaptation fait partie intégrante du système de santé en cas d'anomalie congénitale. Nous estimons que les anomalies congénitales impliquant une réadaptation sont susceptibles de renforcer les systèmes et mesures en la matière grâce à: l'amélioration de la coordination entre les soins primaires et la réadaptation; l'identification et la compréhension des parcours qui permettent aux familles d'entrer en contact avec de tels services; la mise à disposition de ressources humaines compétentes dans ce domaine; et enfin, grâce au développement des mécanismes et ressources qui soutiennent les technologies d'assistance et la réadaptation. Nous proposons aux pays des pistes favorisant un diagnostic précoce, un transfert et une prise en charge des enfants souffrant d’anomalies congénitales, afin de consolider le système de santé et d'en faire bénéficier l'ensemble de la population. En outre, nous recensons les moyens d'étoffer les politiques et programmes, mais aussi d'élaborer des stratégies de prestation de services et de gestion des effectifs conformes aux lignes directrices et cadres relatifs à la réadaptation formulés par l'Organisation mondiale de la Santé. Selon nous, la communauté sanitaire internationale doit agir pour faire en sorte que les services de réadaptation soient pris en compte, acceptés et intégrés dans les systèmes de santé afin de contribuer au bon fonctionnement dès la naissance, et que le handicap figure parmi les priorités en ce qui concerne la santé infantile. De telles actions renforceraient les systèmes de soins de santé, assureraient un développement optimal dès la venue au monde et rendraient la réadaptation accessible à toutes et tous.

## Resumen

Se han hecho progresos considerables para salvar la vida de los niños menores de 5 años. Sin embargo, estos avances no han permitido que todos los niños prosperen, en particular los niños con discapacidades. En este artículo, se describe la creciente prevalencia de la discapacidad entre los niños y adolescentes. Se evalúa la situación actual de los niños con discapacidad y la rehabilitación en el contexto de los sistemas sanitarios, en particular los de los países de ingresos bajos y medios. Dentro de los programas de salud neonatal, las anomalías congénitas suelen requerir una intervención y rehabilitación tempranas. Se menciona a Argentina como ejemplo de un país en donde la rehabilitación de las anomalías congénitas está integrada en el sistema sanitario. Se argumenta que las anomalías congénitas que requieren rehabilitación tienen el potencial de fortalecer los sistemas y las políticas de rehabilitación mediante: el fortalecimiento de la coordinación entre la atención primaria y la rehabilitación; la identificación y la comprensión de las vías que permiten a las familias participar en los servicios; la provisión de recursos humanos para la rehabilitación; y la creación de sistemas y recursos que apoyen la tecnología de asistencia y la rehabilitación. Asimismo, se proponen mecanismos para que los países den prioridad e integren la identificación, derivación y atención tempranas de los niños con anomalías congénitas, con el fin de fortalecer los sistemas sanitarios para todos. Se identifican las oportunidades para ampliar la política y la planificación y para diseñar estrategias de prestación de servicios y de personal a través de las directrices y los marcos de la Organización Mundial de la Salud para la rehabilitación. Se sostiene que la comunidad sanitaria mundial debe actuar para asegurar que los servicios de rehabilitación para apoyar el funcionamiento desde el nacimiento estén bien establecidos, aceptados e integrados dentro de los sistemas sanitarios, y que se dé prioridad a la discapacidad dentro de la salud infantil. Estas medidas reforzarían los sistemas sanitarios, asegurarían el funcionamiento desde el nacimiento y harían que la rehabilitación fuera accesible para todos.

## ملخص

لقد تم إحراز تقدم ملموس في إنقاذ حياة الأطفال دون سن الخامسة. ومع ذلك، فقد فشلت هذه التطورات في مساعدة جميع الأطفال على الازدهار، وبخاصة الأطفال من ذوي الإعاقة. نحن نصف الانتشار المتزايد لحالات الإعاقة بين الأطفال والمراهقين. نحن نقوم بتقييم الوضع الحالي فيما يتعلق بالأطفال من ذوي الإعاقة، وإعادة التأهيل في ظل النظم الصحية، وبخاصة في الدول ذات الدخل المنخفض والدخل المتوسط. في إطار جدول أعمال صحة الأطفال حديثي الولادة، غالبًا ما تتطلب العيوب الخلقية التدخل المبكر وإعادة التأهيل. ونشير إلى الأرجنتين كمثال لدولة تم فيها دمج إعادة تأهيل العيوب الخلقية في النظام الصحي. نحن نناقش موضوع أن العيوب الخلقية التي تتطلب إعادة التأهيل، لديها القدرة على تعزيز أنظمة وسياسات إعادة التأهيل من خلال: تعزيز التنسيق بين الرعاية الأولية وإعادة التأهيل؛ وتحديد وفهم المسارات التي تسمح للأسر بالانخراط في الخدمات؛ وتوفير الموارد البشرية لإعادة التأهيل؛ وبناء النظم والموارد التي تدعم التكنولوجيا المساعدة وإعادة التأهيل. نحن نقترح طرقًا للدول لتساعدها في منح الأولوية وإدماج كل من التحديد المبكر، والإحالة، والرعاية للأطفال الذين يعانون من عيوب خلقية لتعزيز النظم الصحية للجميع. نحن نستكشف الفرص لتوسيع السياسة والتخطيط، وتصميم إستراتيجيات تقديم الخدمات والقوى العاملة، من خلال إرشادات منظمة الصحة العالمية وأطر عمل إعادة التأهيل. نحن نزعم بأن المجتمع الصحي العالمي يجب أن يعمل لضمان أن تكون خدمات إعادة التأهيل لدعم الأداء الوظيفي منذ الولادة، راسخة ومقبولة ومتكاملة في النظم الصحية، وأن يتم منح الأولوية للإعاقة في سياق صحة الأطفال. من شأن هذه الخطوات أن تعزز النظم الصحية، وتضمن الأداء الوظيفي منذ الولادة، وتجعل خدمات إعادة التأهيل في متناول الجميع.

## 摘要

挽救 5 岁以下儿童生命的工作已取得巨大进展。尽管如此，但也未能帮助所有儿童茁壮成长，尤其是残疾儿童。我们描述了儿童和青少年致残率升高的情况。我们评估了卫生体系中残疾儿童及其康复治疗的当前状况（特别针对中低收入国家的儿童）。在新生儿健康议程中，先天性发育异常儿童通常需要尽早接受干预和康复治疗。有些国家已将先天性发育异常儿童的康复治疗纳入国家卫生体系，例如阿根廷。我们认为，针对需要接受康复治疗的先天性发育异常情况，可能可以通过以下方式完善康复系统和相关政策：促使初级保健机构和康复治疗机构之间加强协作；了解和确定允许家庭参与服务的途径；加强康复服务人员队伍建设；以及构建支持辅助技术和康复治疗的系统和资源。我们建议各国通过采取一些手段来优先关注和整合先天性发育异常儿童的早期识别、转诊和护理，并加强构建覆盖全人群的卫生体系。我们根据世界卫生组织的康复指南和框架确定可以通过哪些机会来扩大政策和规划范围，并制定服务提供战略和人才战略。我们认为，全球健康社区都必须采取行动，确保在卫生体系中完善、纳入和整合自出生起即可享受的康复服务，并优先考虑儿童健康问题中的残疾问题。这些步骤将完善卫生体系，确保自出生起即可享受服务，并使所有人都能获得康复治疗。

## Резюме

Значительный прогресс достигнут в спасении жизни детей младше 5 лет. Тем не менее эти достижения не способствуют полноценному развитию всех детей, особенно детей с ограниченными возможностями. В статье рассматривается растущая распространенность инвалидности среди детей и подростков. Оценивается текущая ситуация в отношении детей с ограниченными возможностями и реабилитации в контексте систем здравоохранения, особенно в странах с низким и средним уровнем дохода. В рамках программы охраны здоровья новорожденных врожденные аномалии часто требуют раннего вмешательства и реабилитации. Аргентина приведена в качестве примера страны, где реабилитация при врожденных аномалиях интегрирована в систему здравоохранения. По мнению экспертов, врожденные аномалии, требующие реабилитации, могут способствовать укреплению систем и политики реабилитации за счет усиления координации между первичной медико-санитарной помощью и реабилитацией, определения и понимания путей, позволяющих семьям обращаться за услугами, обеспечения кадровых ресурсов для проведения реабилитации, создания систем и ресурсов, поддерживающих использование технических средств и реабилитации. Для укрепления систем здравоохранения во всех странах предлагается определить приоритеты и интегрировать методы раннего выявления, направления к врачу-специалисту и ухода за детьми с врожденными аномалиями. Выявляются возможности для расширения политики и планирования, а также для разработки стратегий предоставления услуг и кадров на основе руководящих принципов и основных положений Всемирной организации здравоохранения по реабилитации. По мнению экспертов, мировое сообщество здравоохранения должно принять меры для того, чтобы реабилитационные услуги для поддержания функционирования с рождения были хорошо налажены, приняты и интегрированы в системы здравоохранения, а инвалидности уделялось особое внимание в рамках охраны здоровья детей. Эти шаги позволят укрепить системы здравоохранения, обеспечить функционирование с самого рождения и сделать реабилитацию доступной для всех.

## Introduction

There are at least three complementary reasons why countries should urgently prioritize rehabilitation services to promote functioning from birth. First, while remarkable gains in child survival have been made over the past three decades, this progress has exposed a growing inequity. The likelihood of a child having a disability before their fifth birthday was 10 times higher than the likelihood of dying (377 versus 38 per 1000 live births) in 2019,^1^ and the number of children with disabilities is increasing in low- and middle-income countries.^2^ Despite this growing need for rehabilitation services, most health systems lack the capacity and focus to address the needs of children with disabilities^3^ – and an exclusive focus on survival, rather than functioning, overlooks a growing population whose rehabilitation needs have been ignored.

Second, affordable, timely and accessible rehabilitation and assistive technology improves health and well-being, functioning and participation,^3^ and this effect, in many cases, is amplified when started early in life. Early detection, education, intervention, and interdisciplinary coordination for rehabilitation optimizes a child’s quality of life.^4^ The limited evidence available shows major gaps and unmet needs for such services, with 240 million children and adolescents with disabilities lacking access to health services that would support their highest level of functioning.^5^ The long-term implications for socioeconomic development and human capital are profound.^2^^,^^6^

Third, providing access to appropriate care, including rehabilitation, upholds the rights of people with disabilities. Global political support to address inequity in child health is enshrined in the Convention on the Rights of the Child,^7^ the Convention on the Rights of Persons with Disabilities^8^ and the sustainable development goals (SDGs).^9^ SDG 3 to “ensure healthy lives and promote well-being for all at all ages” recognizes rehabilitation as fundamental to universal health coverage. Fulfilling these pledges is important: in signing, governments have committed to provide equitable access to rehabilitation and assistive technology for all individuals, including children.

Despite a large and growing need, provision of rehabilitation and assistive technology is affected by systemic exclusion from global health financing, leadership, and programming relative to investments in other conditions.^10^^,^^11^ Unlike other essential health services, the unmet need for rehabilitation in many low- and middle-income countries exceeds 50%.^10^^,^^12^ Integration of rehabilitation in health services to detect and support children and adolescents with disabilities remains inequitably deficient.^13^ Prioritizing functioning from birth may therefore be a strategic focus to strengthen rehabilitation systems and policies for all.

## Prioritizing rehabilitation

The need to strengthen early intervention and rehabilitation is recognized within the survive and thrive newborn health agenda^14^ to ensure the greatest benefit for millions of babies at risk of preventable long-term impairment. Specifically, congenital anomalies – conditions that involve changes in body structure or function that are present at birth – are a common cause of disability in children and benefit from rehabilitation that starts early. The 2020 World Health Organization (WHO) guidelines on standards for improving quality of care for newborns in health facilities^15^ recommend that all newborns be assessed for congenital anomalies, managed appropriately and referred in a timely manner. This guidance is important because in 2019, congenital anomalies were the 10th most important cause of loss of health globally.^16^ Congenital anomalies affect 6% (60/1000 live births) of the global population, with more than 90% of cases estimated to occur in low- and middle-income countries.^17^ Most congenital anomalies in low- and middle-income countries are underreported and untreated^17^ even though most cases can be improved, managed or treated with appropriate health care.^14^^,^^17^ Early detection and rehabilitation is critical, not only for developmental outcomes^14^ but in determining whether children access rehabilitation at all.^18^

In 2020, WHO published updated guidance for screening and reporting congenital anomalies.^19^ This guidance supports strategies to prioritize and improve service provision for these conditions in health systems. The guidance designates 15 external congenital anomalies defined using three criteria: the conditions are easy to diagnose at birth (requiring no specialized equipment), they have a substantial effect on public health, and there is potential for primary prevention or management.

Among the 15 major external congenital anomalies, three external anomalies are classified as incompatible with life: anencephaly, craniorachischisis and iniencephaly. Three conditions – exomphalos (omphalocele), gastroschisis and hypospadias – require surgical correction only. The remaining nine conditions all benefit considerably from early intervention, rehabilitation and provision of assistive technology (Table 1).

**Table 1:** Prevalence, disability weight and care requirements for nine external congenital anomalies that benefit from early identification and rehabilitation

External congenital anomaly	Description	Birth prevalence, per 10 000 live births^a^	Disability weight, median (range)^b^	Assistive technology requirements^c^	Rehabilitation requirements
Talipes equinovarus (clubfoot)	A fixation of the foot where the foot points downward and inward and is rotated outward axially	10.00–15.00	0.237 (0.163–0.324)^d^	Foot abduction braces to prevent recurrence	The Ponseti method results in a 90% correction rate and is the best practice for clubfoot treatment. Relapse occurs mostly due to non-compliance of bracing, and bracing is recommended for up to 4 years.^20^ The Ponseti method in younger children has improved outcomes compared with surgery. Relapse in surgically treated feet is harder to treat. Conservative treatment is provided by physiotherapists, occupational therapists and other rehabilitation professionals, with specialized intervention by orthopaedic surgeon.^21^
Reduction defects of upper and lower limbs (limb reduction)	The absence or severe hypoplasia of any limb or part of a limb	5.00–7.00	0.15 (0.07–0.24)	Prosthetics or orthotics, mobility aids, self-care aids	Provision of prostheses is guided by clinical experience. Early initiation of rehabilitation and prescription of prosthesis before the age of 2 years has lower rejection rates and higher functional outcomes.^22^ Physiotherapists, occupational therapists, orthotists and prosthetists provide care.^23^
Spina bifida	General term used to describe a neural tube defect of the spine in which part of the meninges or spinal cord or both protrudes through an opening in the vertebral column	0.60–38.9	0.30 (0.01–0.58)	Self-care: body-worn absorbent products (single use or washable); toilet and shower chairs Mobility: wheelchairs and cushions; ankle–foot orthoses; crutches; hand rails and grab bars; portable ramps; rollators; walking frames	Aerobic and strength training improves cardiorespiratory endurance and muscle strength.^24^ Ankle–foot orthosis and crutches improve gait and walking.^25^
Cleft lip and cleft palate	A cleft of the upper lip extending through the hard palate (primary and secondary palate) which may also extend through the soft palate	6.00	0.12 (0.08–0.164)	Communication: boards and books; audiovisual materials	Speech therapy is useful for speech intelligibility^26^ and feeding therapy is beneficial. Clinical guidelines for multidisciplinary treatment for the oral health of children with cleft lip and palate are lacking.^27^
Cleft lip alone	A partial or complete fissure of the upper lip. It can be unilateral or bilateral. The cleft lip can extend through the gum, but not beyond the incisive foramen	3.50	NA		
Cleft palate alone	Characterized by a fissure in the secondary palate; it can involve the soft palate only or both the hard palate and the soft palate. The lip is intact	6.00	NA		
Microcephaly	A cranial vault that is smaller than normal for the infant’s sex and gestational age at birth, and the size of the cranial vault is an indicator of the size of the underlying brain	0.46–5.85	0.32 (0.01–0.63)^d^	Self-care: body-worn absorbent products (single use or washable); toilet and shower chairs Communication: boards and books Mobility: wheelchairs and cushions; crutches; hand rails and grab bars; portable ramps; rollators; walking frames	Long-term follow-up is recommended for all infants with congenital infections including those who appear unaffected. Hearing screening should be undertaken as early as possible to improve language development. Psychosocial support and counselling will be ongoing into adulthood. Early child stimulation may take advantage of the plasticity of the developing brain to improve cognitive outcomes.^28^
Microtia and anotia	A congenital malformation of the ear in which the external ear is either underdeveloped and abnormally shaped (microtia), or absent (anotia). The external ear canal might be absent	0.50–3.30	0.16 (0.01–0.32)^d^	Communication: boards and books; hearing aids and accessories	No evidence is available from systematic reviews. Expert opinion from the International Microtia and Atresia Workgroup was used to develop international consensus guidelines. Treatment of microtia depends on the type and severity of the condition and the age of the child, which will dictate the need and timing of surgery. Surgery and rehabilitation should be decided on and managed with a team including otolaryngologist/otologist, hearing specialist and/or audiologist, speech therapist and school specialists.^29^
Encephalocele	Characterized by a pedunculated or sessile cystic, skin-covered lesion protruding through a defect in the skull bone	0.10–26.5	0.29 (0.01–0.56)	Self-care: body-worn absorbent products (single use or washable); toilet and shower chairs Communication: boards and books Mobility: wheelchairs and cushions; crutches; hand rails and grab bars; portable ramps; rollators; walking frames	No evidence is available from systematic or literature reviews.

NA: not applicable.

^a^ Source: World Health Organization, 2020.^19^

^b^ Source: Institute for Health Metrics and Evaluation, 2019.^30^

^c^ Source: World Health Organization, 2016.^31^

^d^ Where the Global Burden of Disease collaboration has yet to assign a specific disability weight for a condition, a range for similar characteristics was used as a substitute.^32^

Systems and policies to support rehabilitation in children with congenital anomalies do not have to be at odds with providing health care for all. Moreover, they may provide an entry point to make rehabilitation accessible to all. Clubfoot and limb reduction are good examples of congenital anomalies that are easily recognizable and detected at birth, and require early intervention to support functioning. Rehabilitation and assistive technology can result in substantial, sometimes complete, improvement of function for children with clubfoot^20^^,^^21^ and limb reduction.^22^^,^^23^ Both conditions require service provision by physiotherapists or similar types of rehabilitation professionals. The conditions also require a series of assistive products to be worn over years to support functioning. Management of both these conditions requires regular follow-up with a health provider, allowing regular contact for children with the health system, and providing opportunities to address any other emerging health issues. This interaction offers the possibility for more holistic care for children and adolescents as they grow and develop.

Strengthening the provision of rehabilitation services for such conditions within primary health-care clinics and centres builds capacity that will be useful for all individuals who may benefit from rehabilitation services. The systems and resources supporting assistive technology for these two conditions are similar for most birth anomalies, injuries and childhood disabilities. Therefore, the shared challenges and benefits of addressing clubfoot and limb reduction can be extrapolated to numerous childhood conditions to build appropriate models of care in low- and middle-income countries.^33^

## The case of Argentina

Argentina is an example of a country that has prioritized systems for integrating early identification, referral and care for children with congenital anomalies. The National Network of Congenital Anomalies of Argentina started in November 2009 in four provinces in Argentina as a hospital-based registry.^34^ The Network expanded its objectives from finding causal factors and generating and disseminating epidemiological information about the prevalence of congenital anomalies^35^ to improving care for affected newborns.^36^ The systems-level impact of Argentina’s integrated rehabilitation has resulted in multifaceted benefits for newborns and older children, their families and service providers. The Network collaborates with, and supports, the main public and private maternity hospitals of the 24 jurisdictions of the country covering about 300 000 births a year.^37^ This coverage equates to 62% (300 000/483 871) of births in the public sector and 43% (300 000/697 174) of births in Argentina.^36^ This outcome was achieved through adopting a comprehensive systems-level approach, despite a context where health systems are fragmented and some health services are scarce.^38^ For instance, the Network is the coordinating centre for newborns identified with cleft lip and cleft palate in partnership with the Sumar programme.^36^ The Sumar programme of the health ministry emphasizes preventive health-care services for uninsured people, while mandating predefined quality standards for the services provided; the programme pays provinces based on their performance against specific health goals.^36^ A toolkit for the health care needs assessment, the Public Health and Genomics Foundation toolkit^39^ supported Argentina’s needs assessment and the development of services and interventions for children with congenital anomalies.^38^ Among other activities, health professionals from different levels of care, with an emphasis on primary care, are supported through training programmes^38^ and information collected by the Network is disseminated to stakeholders, including the participating clinicians who are empowered to use their own processed data. The outcomes of this systems-level approach include: an increase in numbers of affected children being referred to services each year; informed and engaged public and parents; increased numbers of trained health-care providers; and policy-makers assisted to implement services for care and prevention programmes.^36^^,^^40^ Challenges in integrating rehabilitation into the health system have included coordination of multiple stakeholders and meeting the demand of the population for genetic diagnosis and counselling.^38^

## The way forward 

We foresee several ways for countries to prioritize and integrate early identification and referral of and care for children with congenital anomalies, and thus to strengthen health systems for all.

Identifying children with congenital anomalies within the primary care system and referring them for care would strengthen coordination between primary care and rehabilitation. This approach would also address policy and leadership gaps for rehabilitation at health ministries and in health systems governance.^41^ Nurses, midwives, skilled birth attendants and community health workers need to be trained in their roles to: (i) recognize conditions at birth; (ii) provide accurate parent education on congenital anomalies which may reduce stigma, misinformation and risks to the child of abuse or neglect;^14^ and (iii) refer children for rehabilitation. Their role strengthens the important pathway for all impairments diagnosed in infancy and early childhood, and the capacity developed will not only benefit children with these conditions, but others as well.

At the community level, stigma, negative attitudes of caregivers and health-care providers, and a lack of knowledge and skills among health workers are barriers to early intervention.^12^^,^^42^ Identifying and understanding the pathways that allow families to engage with services, and the barriers they encounter, will inform the planning of accessible rehabilitation. One example may be to design education resources with caregivers, who can give unique insights about their challenges and situations.^43^ In turn, caregivers can be appropriately informed and engaged with services. Likewise, building capacity and expertise and providing human resources for rehabilitation can be achieved through promoting education of health-care providers, as in the case of Argentina. Increasing the sensitization and education of health workers can result in higher rates of referral and better information given to parents at the point of referral.

Strengthening multidisciplinary coordination for congenital anomalies can improve the health system functioning for all. When rehabilitation is prioritized from birth, as is indicated for clubfoot and limb reduction, for example, these multidisciplinary connections are enhanced, as rehabilitation services are coordinated across several care teams. For clubfoot, the Ponseti method may require a surgeon to perform an outpatient Achilles tenotomy or to address recurrence;^44^ in limb reduction, conversion amputation may be necessary.^45^ Coordination between surgery and post-surgical rehabilitation can substantially improve outcomes of surgical intervention^42^ and such coordination is relevant to other childhood disabilities including cerebral palsy, spina bifida and trisomy 21.

Strengthening systems and resources that support rehabilitation and assistive technology at the policy level will assist policy-makers in implementing services for care and rehabilitation. Currently, the absence of appropriate assistive technology supply chains, political will and global investment limits rehabilitation efforts; only an estimated 5–15% of people who need assistive technology can access it in low- and middle-income countries.^46^ National policies and regulations are required to provide an enabling environment to ensure a functioning supply chain of essential equipment for children with congenital abnormalities, which will benefit all users of assistive technology.

Opportunities exist to expand planning for rehabilitation through WHO guidelines and frameworks, which shape governments’ health expenditure, development assistance and targets. For example, in the absence of adequate government-led investment in rehabilitation in low- and middle-income countries, WHO’s Rehabilitation 2030^47^ should prioritize strategic entry points, such as newborn and child health, to activate health-sector awareness and adaption of rehabilitation in primary care. A focus on neonatal and paediatric rehabilitation would strengthen high-level multidisciplinary coordination between WHO leadership for maternal, newborn, child and adolescent health, human resources for health,^48^ essential surgical and trauma care, Rehabilitation 2030^47^ and the Global Cooperation on Assistive Technology.^49^ Another example is the 2020 WHO birth defects surveillance manual^19^ that filled a critical need by addressing information gaps in systematic surveillance. One way to accelerate progress beyond surveillance would be to provide comprehensive evidence-based care guidance in Rehabilitation 2030’s package of interventions,^47^ aligned with the WHO- and United Nations Children’s Fund-led survive and thrive plan^14^ for transforming newborn outcomes.

For referral systems to function, they require national data, formalized roles, supervision and leadership, and targets. In Argentina, reporting into centrally managed open source medical records systems allows patients to be tracked and trends in treatment to be monitored over time. Adequate record-keeping is essential to identify children who are being lost to follow-up. While success of treatment can be determined through analysis of documented clinical findings, complex paperwork systems or the lack of a recording system make central analysis and identification of individual clinic results difficult. Rehabilitation practitioners may be motivated to record data that are seen as immediately beneficial, and identification of simple processes to collect these data is needed. Data are also vital for monitoring service availability and quality. Integrating the Rehabilitation 2030 service delivery indicators^50^ into other service delivery frameworks will inform health programme managers as they design, monitor and evaluate primary care rehabilitation through centralized national-level reporting. These data can be used to inform the number and rational distribution of clinics and health workers providing rehabilitation and assistive technology services.

Gaps remain in the health-policy and systems-research agenda to prioritize functioning from birth, and these gaps present opportunities for future research. More widespread and nuanced evidence of systems approaches to managing common congenital and childhood causes of impairment in low- and middle-income countries is essential to inform national rehabilitation plans that guide efforts to make these services accessible from birth. Valid, reliable and timely data are essential for priority-setting, planning and delivery of high-quality rehabilitation services to all. For example, accurate national birth prevalence data and population estimates of congenital anomalies are needed for strategic planning and proportionate resource allocation for rehabilitation and assistive technology services. These data require enhanced country-level information systems to support complete and accurate reporting of newborn and child health indicators on functioning and impairments. These enhancements, in turn, will link rehabilitation to broader improvements in national health systems and health information systems. Likewise, reporting on and monitoring rehabilitation-specific professionals in national human resources information systems is vital to inform workforce development and distribution. Global evidence-based guidelines for the treatment and rehabilitation of children with congenital anomalies are also needed to strengthen the quality of service provision. Data on coverage and access to rehabilitation services for children with congenital anomalies, as well as other social determinants of health and equity measures, need to be included at local levels of health systems to ensure performance management for equity and to inform rehabilitation and assistive technology provision. Integrating these data with routine health information systems and ensuring the data are made accessible and actionable for national and subnational health leadership is essential to strengthen rehabilitation services from birth.

## Conclusion

Rehabilitation needs a focus to move beyond rhetoric to strategy and harness political will and resources. A strategy that prioritizes identifying and supporting babies with congenital anomalies can strengthen rehabilitation and assistive technology delivery for children and adolescents with other conditions. A rights-based approach is vital. This approach requires a fundamental shift in understanding that the functioning of an individual is central to population health and to society, and equal in benefit to preventing disability and death. By prioritizing disability in child health, governments and global agencies can support the health system infrastructure required to enhance rehabilitation and disability-inclusive services for all. 
